# The association of relational and organizational job stress factors with sleep disorder: analysis of the 3rd Korean working conditions survey (2011)

**DOI:** 10.1186/s40557-016-0131-2

**Published:** 2016-09-13

**Authors:** Gyuree Kim, Bokki Min, Jaeyoup Jung, Domyung Paek, Sung-il Cho

**Affiliations:** Department of Occupational and Environmental Medicine, Graduate School of Public Health, Seoul National University, Gwanak-ro 1, Gwanak-gu, Seoul 151-600 Republic of Korea

**Keywords:** Job-related stress factor, Sleep disorder, KWCS

## Abstract

**Background:**

Sleep disorder is a disease that causes reduction in quality of life and work efficiency of workers. This study was performed to investigate the relationship between job-related stress factor and sleep disorder among wageworkers in Korea.

**Methods:**

This study was based on analysis of the 3rd Korean working conditions survey. We analyzed 35,902 workers whose employment status is wageworker. We classified the job-related stress factor into 12 sections. Logistic regression was performed to estimate the relationship between job-related stress factor and sleep disorder and Odds ratio and 95 % CI were calculated using the SPSS version 23.0 program.

**Results:**

Many categories of Job-related stress factor were correlated with sleep disorder (8 of 12 for women, 10 of 12 for men). The results of the regression analysis, corrected for general and occupational characteristics, indicated that sleep disorder was significantly correlated with the following categories of job-related stress: discrimination experience (OR 3.37, 95 % CI = 2.49 ~ 4.56 in women, OR 1.96, 95 % CI = 1.53 ~ 2.51 in men), direct customer confrontation (OR 2.72, 95 % CI = 1.91 ~ 3.86 in women, OR 1.99, 95 % CI = 1.45 ~ 2.72 in men), emotional stress (OR 2.01, 95 % CI = 1.30 ~ 3.09 in men), work dissatisfaction (detailed) (OR 1.99, 95 % CI = 1.36 ~ 2.93 in men), work dissatisfaction (overall) (OR 2.30, 95 % CI = 1.66 ~ 3.20 in women, OR 2.40, 95 % CI = 1.88 ~ 3.08 in men), expression of opinion difficulty (OR 0.66, 95 % CI = 0.48 ~ 0.92 in women, OR 0.57, 95 % CI = 0.45 ~ 0.73 in men).

**Conclusion:**

A number of studies have reported that stress affects sleep disorder. In this study, many factors suspected to increase the risk of sleep disorder were added to previously known job stress factors. In particular, this study found a strong correlation between work-associated sleep disorder and relational and organizational job stress factors. Sleep disorder may lead to large decreases in workers’ quality of life and work efficiency. Awareness and interventions are therefore required to reduce workplace stress; additional research of this topic is also required.

**Electronic supplementary material:**

The online version of this article (doi:10.1186/s40557-016-0131-2) contains supplementary material, which is available to authorized users.

## Background

Research has increasingly examined means of improving workers’ health and the working environment, in accordance with the increasing popularity of social change for improved welfare. National-level efforts to manage workers’ health and establish pleasant working environments have emerged; for example, the enactment of the Industrial safety and health act by the Ministry of Employment and labor [1], in addition to personal health management.

Psychiatric problems such as sleep disorder, neurological headaches, and depression are often considered personal problems rather than job-associated problems, and therefore receive less attention than relatively serious occupational health problems such as suicide, physical injury, and accidents [2]. Nonetheless, the International Classification of sleep Disorders (ICSD; the most widely used sleep disorder classification) nominates stress as the primary cause of sleep disorders: relationship stress or decreased psychosocial abilities weaken physical resilience and psychological integrity [3]. Further, occupational risk factors (e.g., shift work, job-related stress) are related to insomnia [4].

The ICSD-3, a standard for ICSD diagnosis, classifies general sleep disorders including insomnia into seven categories: 1) insomnia, 2) sleep-related breathing disorders, 3) central disorders of hypersomnolence, 4) circadian rhythm sleep-wake disorders, 5) parasomnias, 6) sleep-related movement disorders and 7) other sleep disorders [5]. Of these, insomnia (sleep abnormalities that persist despite suitable opportunities and environment) is the most common of the sleep disorders. Its prevalence is high worldwide (10–30 % in western countries; 15–21 % in Asian countries [6–11]), suggesting that the total prevalence of all sleep disorders is considerably higher. Further, regarding South Korea, 17 % of the population reports experiencing insomnia ≥3 times a week [12] and 31 % of the general adult population reports sleep problems such as difficulty falling asleep, frequent waking, and early morning awakening [13]. Further, long-lasting sleep disorders predict reduced productivity and work efficiency among workers, ultimately leading to decreased company productivity. Sleep disorder increases the risk of reduced workers’ well-being and quality of life; sleep disorder therefore presents a serious problem that requires attention.

Jang et al. developed the Occupational Stress Scale for Korean Workers; Kim et al. used that scale to examine the correlation between occupational stress and sleep disorders. They found that individuals experiencing higher job stress experience insomnia more frequently [14]. Heo et al. found that physical factors and psychosocial factors increase individuals’ risk of sleep disturbance; they examined data from the 2nd Korean Working Conditions Survey (KWCS) [15].

This study analyzed job-related stress’ effects on sleep disorder, as addressed in previous studies, as well as factors newly identified to affect sleep disorder (e.g., discrimination experience and emotional labor), using data from the third KWCS. It is possible that workers, especially in the rapidly increasing service industries like telemarketing, the insurance business, etc., experience stress in their relationships with customers. Furthermore, labor experiences and efficiency may be affected by organizational culture and structure, as well as relationships between individuals because of the generalization of production systems per unit, module, and team. For this reason, we focused on relational and organizational job stress factors.

Our analysis extends the understanding of sleep disorders to include correlations with novel factors. Additionally, in contrast to earlier research we analyzed the interactions between gender and each job stress factor, and we presented the results of the analyses separately by gender. Therefore, this article clearly demonstrates the gender differences in exposure and risk of stress and disease. Furthermore, this analysis addressed the associations between work and sleep disorders, thereby examining job-related factors’ effects on the development of occupational diseases.

## Methods

### Study subjects

This study used data from the KWCS, which was conducted in 2011 by the Occupational Safety and Health Research Institute (OSHRI) of the Korea Occupational Safety and Health Agency (KOSHA). The KWCS examined overall conditions of employment (form of labor, form of employment, occupation, industry, exposure to risk factors, and stability) in employees aged ≥15 years in Korea. The KWCS selected households from the 2005 Population and Housing Census; individuals who met criteria defining an “employee” received one-on-one interviews from a professional interviewer who visited their house. The Statistics Korea accredited the KWCS’ reliability in order to increase the usage of the data it collected: the survey’s response rate was 0.354, the cooperation rate was 0.662, and the refusal rate was 0.180 [16]. The final sample size was 50,032 individuals; 35,902 of whom were employed workers who received wages. These individuals were finally selected for examination in the present study after excluding non-wage workers such as self-employed without employees or self-employed with employees. Weighted statistical analysis was performed to prevent bias and holistically represent working conditions in Korea.

## Variables (Methods)

### 1. Independent variables

#### General characteristics

Following the KWCS, factors relating to general employee characteristics (e.g., sex, age, education, income, alcohol and smoking) were classified as independent variables. Each variable was defined based on survey contents. Age was divided into the following groups: 15–29, 30–39, 40–49, 50–59, and ≥60 years. Education was categorized into the following groups: middle school completion or less, high school completion, and college completion or above. Monthly income was categorized into the following groups: ≤990,000 Korean won, 1,000,000–1,990,000 won, 2,000,000–2,990,000 won, and ≥3,000,000 won. Alcohol (drinking habits) was categorized into the following groups: none, once or less per week, and twice or more per week. Smoking was categorized into the following groups: non-smoker, ex-smoker, and current-smoker.

#### Occupational characteristics

Following the KWCS, factors reflecting occupational characteristics were classified as occupational independent variables (e.g., occupation, work type, working hours per week, tenure, shift work, and workplace scale). Variables were defined based on the KWCS’s contents. Occupations were categorized into the following groups: white-collar workers (professional and technical occupations, higher administrator occupations, clerical occupations, sales occupations), service workers, and blue-collar workers (skilled, semi-skilled, unskilled and farm workers). Work type was categorized into either regular positions or temporary or part-time positions. Working hours per week were categorized into two groups with 45 h as the standard. Tenure (number of years worked) was categorized into two groups with 1 year as the standard. Workers were categorized as shift or non-shift workers; workplace scale was categorized into the following groups based on the number of workers they employed:≤99, 100–999, ≥1000, and unknown.

### Job-related stress factor

Job-related stress was sorted into the following 12 categories: high job demand, insufficient job control, inadequate social support, job insecurity, lack of reward, discrimination experience, direct customer confrontation, emotional stress, work dissatisfaction (detailed), work dissatisfaction (overall), organizational system dissatisfaction and expression of opinion difficulty.

This categorization followed the Occupational Stress Scale for Korean Employees [17], which supported eight categories of occupational stress, and on previous research that has supported seven and five categories reflecting job stress and psychosocial factors, respectively [14, 15]. These categories were collectively organized and compared to the KWCS to confirm that the selected items addressed each category. Five categories were subsequently selected following commonly examined variables; seven additional categories were selected following examination of items addressing relational and organizational job stress factors in the KWCS.

Commonly examined variables from earlier research were organized into the following five categories: high job demand (seven questions: very fast-paced work, working under strict deadlines, needing to halt in order to complete unforeseen work, strict quality standards, needing to self-assess the quality of one’s work, needing to solve unforeseen problems by oneself, sufficient time given to complete work), insufficient job control (seven questions: ability to spend 1–2 h during work hours to run personal errands, ability to change order of work, ability to change method of work, ability to change speed of work, ability to choose a co-worker, ability to take a break when desired, ability to influence important decisions at work), inadequate social support (eight questions: support from co-workers, support from superiors, feedback from superiors regarding one’s work, superiors conflict-resolution ability, respectful treatment from superiors, superiors’ planning and organizational ability, encouragement from superiors to participate in important decision-making), job insecurity (two questions: loss of job within the next 6 months, ability to find a job with a similar wage in case of unemployment) and lack of reward (one question: proper compensation provided for work).

Questions examining novel variables were organized into the following seven categories: discrimination experience (five questions: discrimination by gender, age, education level, region and employment type), direct customer confrontation (two questions: Dealing directly with people who are not employees at your workplace, Handling angry clients), emotional stress (three questions: get emotionally involved in work, experience stress in work, Your job requires that you hide your feelings), work dissatisfaction (detailed) (five questions: Your job gives you the feeling of work well done, You are able to apply your own ideas in your work, You have the feeling of doing useful work, You know what is expected of you at work, Your job involves tasks that are in conflict with your personal values), work dissatisfaction (overall) (one question: satisfied with working conditions on the whole), organizational system dissatisfaction (three questions: My job offers good prospects for career advancement, I feel ‘at home’ in this organization, The organization I work for motivates me to give my best job performance) and expression of opinion difficulty (two questions: You are consulted before targets for your work are set, You are involved in improving the work organization or work processes of your department or organization).

The novel variables were added to the variables drawn from earlier research and analyzed. Responses to each question were divided based on the median score and summed; they were then categorized into high risk and low risk groups. Regarding responses to high job demand, Cronbach’s alpha was 0.600, that of insufficient job control was 0.658, inadequate social support was 0.719, discrimination experience was 0.619, emotional stress was 0.692, work dissatisfaction (detailed) was 0.727, and organizational system dissatisfaction was 0.655. Cronbach’s alpha was not calculated for responses to categories containing only one or two questions (i.e., job insecurity, lack of reward, direct customer confrontation, work dissatisfaction (overall), expression of opinion difficulty).

### 2. Dependent variables

Presence or absence of sleep disorder was the dependent variable. Of the individuals who responded “yes” to the KWCS question “Over the last 12 months, did you suffer from insomnia or general sleep difficulties?,” those who answered “yes” to “if yes, Are your health problems due to your job?” were classified as having sleep disorder. Health problems’ relationship with work was measured, in addition to sleep disorder’s presence or absence, in order to improve the representation of the association between sleep disorder and work.

### Statistical analysis

A Chi-square test was used to examine sleep disorder’s relationship with the existing and additional research variables. Additionally, multivariable logistic regression analyses that corrected for all general and occupational variables were carried out. These analyses were performed separately and were divided by gender. Statistical significance was set at *p* < 0.05; all analyses were performed using SPSS v.23.0.

## Results

### General characteristics and occupational characteristics of study subjects

59.3 % of participants were male (21,285 individuals); 40.7 % were female (14,616 individuals). 15.6 % of participants were aged ≤29 years, 30.6 % were aged 30–39, 29.2 % were aged 40–49, 16.9 % were aged 50–59, and 7.8 % were aged ≥60 years; the most common age group was thus 30–39 years. The most common income group was 1,000,000–1,990,000 Korean won per month (37.7 %), more than half of participants had completed a college qualification or higher. Around half of the sample consumed alcohol once per week or less (50.8 %); a small majority were non-smokers (54.8 %).

Participants’ most frequent occupation categories were as follows: white collar (48.3 %), blue collar (34.2 %), and service sector (17.5 %). Most employees were regular employed (79.5 %) and 20.5 % held temporary or part-time positions, giving a ratio of 4:1 between these work types. More than half of participants worked overtime (over 45 working hours per week); most participants had worked with their current employer for 1 year or longer. One tenth of participants indicated that they were shift workers (9.4 %). Most participants worked in businesses with 99 employees or less (81.5 %; Table 1).

**Table 1:** General and Occupational Characteristics of study subjects by sleep disorder

Characteristics	N	%	Sleep Disorder		*p*-value
			Yes (%)	No (%)	
Sex					
Female	14616	40.7	217 (1.5)	14399 (98.5)	0.004
Male	21285	59.3	401 (1.9)	20884 (98.1)	
Age (year)					
15–29	5588	15.6	72 (1.3)	5516 (98.7)	0.049
30–39	10971	30.6	202 (1.8)	10769 (98.2)	
40–49	10488	29.2	174 (1.7)	10314 (98.3)	
50–59	6058	16.9	119 (2.0)	5939 (98.0)	
60–	2796	7.8	51 (1.8)	2745 (98.2)	
Occupation					
White collar	17343	48.3	332 (1.9)	17011 (98.1)	0.021
Service worker	6279	17.5	92 (1.5)	6187 (98.5)	
Blue collar	12280	34.2	194 (1.6)	12086 (98.4)	
Income (won)					
− 990,000	4149	11.6	64 (1.5)	4085 (98.5)	<0.001
1,000,000–1,990,000	13534	37.7	198 (1.5)	13336 (98.5)	
2,000,000–2,990,000	10086	28.1	174 (1.7)	9912 (98.3)	
3,000,000–	8133	22.7	182 (2.2)	7951 (97.8)	
Education					
Middle school or below	3893	10.8	74 (1.9)	3819 (98.1)	0.743
High school	13155	36.6	207 (1.6)	12948 (98.4)	
College or above	18853	52.5	337 (1.8)	18516 (98.2)	
Work type					
Regular	28542	79.5	504 (1.8)	28038 (98.2)	0.232
Temporary or part-time	7360	20.5	115 (1.6)	7245 (98.4)	
Alcohol					
No	8215	22.9	164 (2.0)	8051 (98.0)	0.373
≤ Once per week	18230	50.8	284 (1.6)	17946 (98.4)	
≥ Twice per week	9457	26.3	170 (1.8)	9287 (98.2)	
Smoking					
Non-smoker	19613	54.8	308 (1.6)	19305 (98.4)	0.295
Ex-smoker	4037	11.2	105 (2.6)	3932 (97.4)	
Current-smoker	12252	34.1	205 (1.7)	12047 (98.3)	
Working hours per week (hours)					
≤ 45 h	16173	45	216 (1.3)	15957 (98.7)	<0.001
> 45 h	19729	55	403 (2.0)	19326 (98.0)	
Tenure (years)					
≥ 1 year	30672	85.4	527 (1.7)	30145 (98.3)	0.911
< 1 year	5230	14.6	91 (1.7)	5139 (98.3)	
Shift work					
No	32534	90.6	477 (1.5)	32057 (98.5)	<0.001
Yes	3368	9.4	142 (4.2)	3226 (95.8)	
Workplace scale (person)					
~ 99	29253	81.5	434 (1.5)	28819 (98.5)	<0.001
100 ~ 999	4131	11.5	117 (2.8)	4014 (97.2)	
999~	1354	3.8	52 (3.8)	1302 (96.2)	
Unknown	1163	3.2	15 (1.3)	1148 (98.7)	
Total	35902	100.0	618 (1.7)	35284 (98.3)	

*P*-value of chi-squared Pearson’s test

### Association between characteristics and sleep disorder

Six hundred and eighteen participants (1.7 %) indicated experiencing sleep disorder. The rate of sleep disorder was significantly higher among men, among participants aged 50–59, and among participants who earned over 3,000,000 Korean won per month. Slight differences in rate of sleep disorder were correlated with education, alcohol and smoking; however, these were not statistically significant.

The prevalence of sleep disorder was significantly higher among participants with white-collar, those who worked over 45 h per week, shift workers, and those working in businesses with 1000 or more employees. Slight differences in prevalence were correlated with work type and tenure; however, these were not statistically significant (Table 1).

### Relationship between job-related stress factor and sleep disorder

The results of the Chi-square test indicated that both men and women in the high risk groups, including those with high job demand, lack of rewards, discrimination experiences, direct customer confrontations, emotional stress, work dissatisfaction (overall), and organizational system dissatisfaction, more commonly experienced sleep disorders (all differences significant). Alternatively, there were some gender differences seen in terms of those who experienced insufficient job control, inadequate social support, job insecurity, work dissatisfaction (detailed), and expression of opinion difficulty (Table 2).

**Table 2:** Relationship between job-related stress factor and sleep disorder by gender

Characteristics	N	%	Female (*N* = 14616)		*p*-value	Male (*N* = 21285)		*p*-value
			Yes (%)	No (%)		Yes (%)	No (%)	
High job demand					<0.001			0.019
Low	17671	49.2	85 (1.1)	7675 (98.9)		164 (1.7)	9748 (98.3)	
High	18230	50.8	133 (1.9)	6724 (98.1)		238 (2.1)	11136 (97.9)	
Insufficient job control					0.020			0.097
Low	17914	49.9	121 (1.7)	6883 (98.3)		222 (2.0)	10688 (98.0)	
High	17988	50.1	96 (1.3)	7517 (98.7)		179 (1.7)	10196 (98.3)	
Inadequate social support					0.170			0.071
Low	19725	57.7	98 (1.3)	7359 (98.7)		216 (1.8)	12052 (98.2)	
High	14459	42.3	100 (1.6)	6172 (98.4)		173 (2.1)	8014 (97.9)	
Job insecurity					0.431			0.017
Low	14067	39.2	87 (1.4)	6129 (98.6)		125 (1.6)	7726 (98.4)	
High	21835	60.8	131 (1.6)	8270 (98.4)		276 (2.1)	13159 (97.9)	
Lack of reward					<0.001			<0.001
Low	24327	67.8	118 (1.2)	9701 (98.8)		208 (1.4)	14299 (98.6)	
High	11575	32.2	99 (2.1)	4698 (97.9)		193 (2.8)	6585 (97.2)	
Discrimination experience					<0.001			<0.001
No	31487	87.7	133 (1.1)	12528 (98.9)		305 (1.6)	18517 (98.4)	
Yes	4419	12.3	84 (4.3)	1871 (95.7)		97 (3.9)	2367 (96.1)	
Direct customer confrontation					<0.001			<0.001
Low	33212	92.5	166 (1.3)	13056 (98.7)		346 (1.7)	19644 (98.3)	
High	2691	7.5	51 (3.7)	1344 (96.3)		55 (4.2)	1241 (95.8)	
Emotional stress					<0.001			<0.001
Low	6054	16.9	18 (0.7)	2560 (99.3)		23 (0.7)	3453 (99.3)	
High	29849	83.1	199 (1.7)	11840 (98.3)		378 (2.1)	17431 (97.9)	
Work dissatisfaction (detailed)					0.083			<0.001
Low	5585	15.6	22 (1.1)	2057 (98.9)		30 (0.9)	3475 (99.1)	
High	30317	84.4	195 (1.6)	12342 (98.4)		371 (2.1)	17409 (97.9)	
Work dissatisfaction (overall)					<0.001			<0.001
Low	26447	73.7	118 (1.1)	10922 (98.9)		213 (1.4)	15193 (98.6)	
High	9456	26.3	99 (2.8)	3477 (97.2)		188 (3.2)	5691 (96.8)	
Organizational system dissatisfaction					0.008			<0.001
Low	30062	83.7	168 (1.4)	12105 (98.6)		306 (1.7)	17483 (98.3)	
High	5840	16.3	49 (2.1)	2294 (97.9)		95 (2.7)	3402 (97.3)	
Expression of opinion difficulty					0.061			0.002
Low	16023	44.6	103 (1.7)	5925 (98.3)		216 (2.2)	9779 (97.8)	
High	19879	55.4	114 (1.3)	8474 (98.7)		185 (1.6)	11105 (98.4)	
Total	35902	100.0	217 (1.5)	14399 (98.5)		401 (1.9)	20884 (98.1)	

*P*-value of chi-squared Pearson’s test

The logistic regression analyses for the correlations between the additional variables (which are the main focus of this study) and sleep disorders were performed separately by gender, correcting for both general and occupational characteristics. The groups at high risk of discrimination experiences, direct customer confrontations, and work dissatisfaction (overall) showed significantly higher risk of sleep disorders compared to the low risk groups, and this effect was reversed regarding the expression of opinion difficulty for both men and women. For emotional stress and work dissatisfaction (detailed), there were also sleep disorder risks, although with slight differences in 95 % confidence intervals. In particular, the OR values of discrimination experiences among women were much higher than those of men (Table 3).

**Table 3:** Odds ratio of selected variables associated with sleep disorder by gender

Variable	Female		Male	
	OR	95 % CI	OR	95 % CI
Discrimination experience	3.37	2.49 ~ 4.56	1.96	1.53 ~ 2.51
Direct customer confrontation	2.72	1.91 ~ 3.86	1.99	1.45 ~ 2.72
Emotional stress	1.67	0.98 ~ 2.83	2.01	1.30 ~ 3.09
Work dissatisfaction (detailed)	1.03	0.64 ~ 1.65	1.99	1.36 ~ 2.93
Work dissatisfaction (overall)	2.30	1.66 ~ 3.20	2.40	1.88 ~ 3.08
Organizational system dissatisfaction	0.87	0.57 ~ 1.33	1.06	0.79 ~ 1.42
Expression of opinion difficulty	0.66	0.48 ~ 0.92	0.57	0.45 ~ 0.73

Each variables’ reference value is ‘Low’ (‘No’ for ‘Discrimination experience’)

Multivariable logistic regression analysis, correcting other variables (age, occupation, income, working hours, shift work, workplace scale and existing job stress factors)

By incorporating gender interaction, we were able to provide Additional file 1. Crude OR values obtained by univariable logistic regression analysis were calculated to examine sleep disorder’s correlation with each new variable (Model 1). Additionally, multivariable logistic regression analysis that simultaneously analyzed the existing and new variables (Model 2) and analysis that corrected for all general and occupational variables (Model 3) were conducted. Individual ORs and 95 % CIs may be found in the Additional file 1.

## Discussion

This study found that job-related stress factors generally increased the risk of sleep disorders. In particular, discrimination experience, and variables related to emotional labor and overall satisfaction showed OR values of over 2.00, even after correcting for all related variables. This, then, is a strong correlation. Further, the results of the Hosmer Lemeshow goodness-of-fit test measurements for each logistic regression were 0.217 for women and 0.308 for men, both of which were > 0.05 and were therefore adequate. To rule out the possibility of multicollinearity, we assessed the variance inflation factor (VIF), which is an index measuring how much the variance of an estimated regression coefficient increases because of collinearity. A VIF value greater than 10 is considered to indicate high multicollinearity [18]. All the variables in our analyses showed adequate VIF values, since they were smaller than 1.5.

**Fig. 1:**
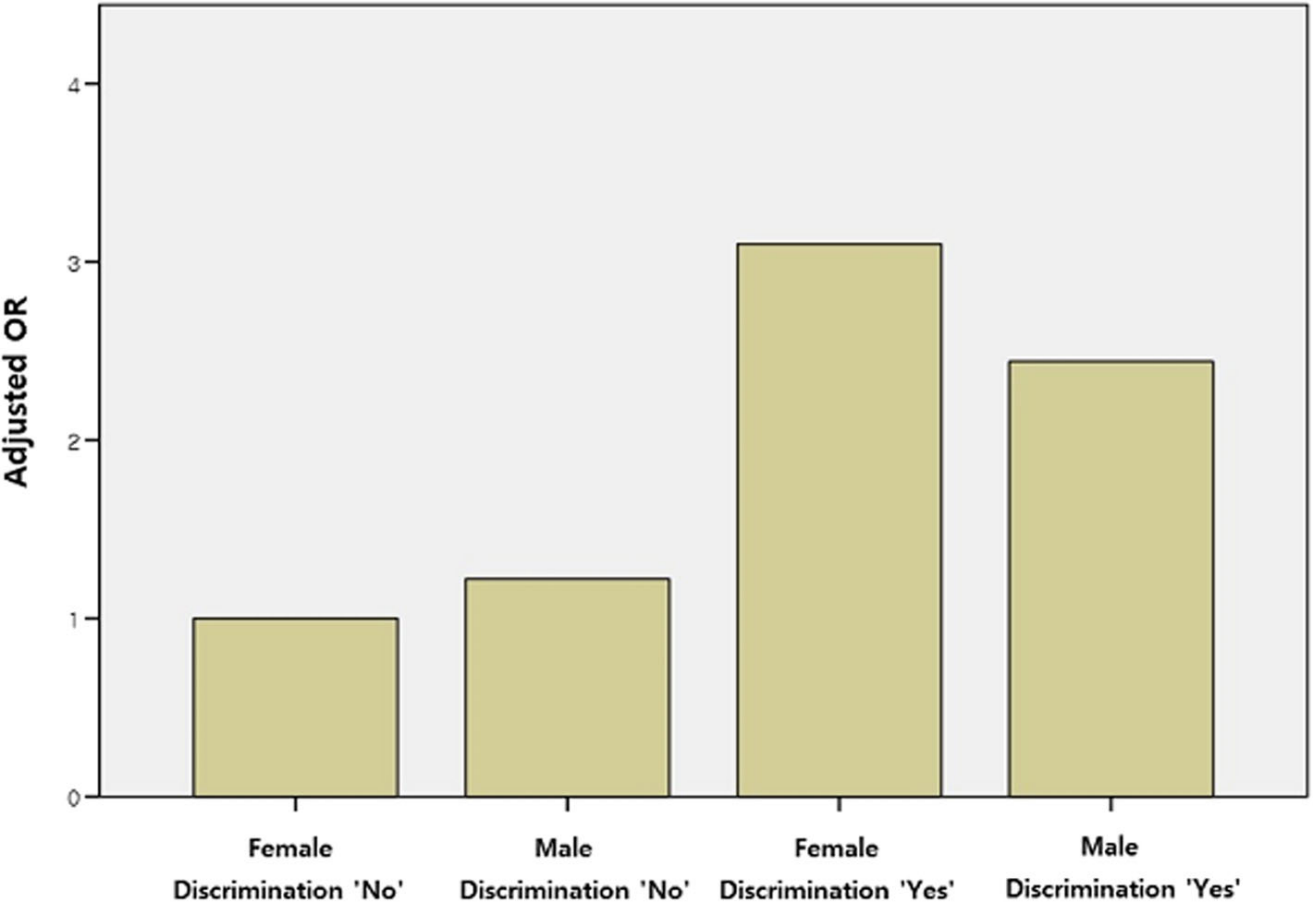
Adjusted ORs for sleep disorder in terms of interactions with gender and discrimination experiences; Adjusted ORs for sleep disorder correcting other variables indicate Female*Discrimination ‘No’ = 1.00, Male*Discrimination ‘No’ = 1.22, Female*Discrimination ‘Yes’ = 3.10, and Male*Discrimination ‘Yes’ = 2.44

Because there are reportedly gender differences in stress exposure and risk [19, 20], we analyzed the interactions between gender and each job stress factor. Discrimination experience was significantly correlated with gender (and the effect of discrimination on sleep disorders was larger among women than men, as seen in Fig. 1); however, no other significant interactions were found. Therefore, we performed the regression analysis separately by gender, and found that the OR and statistical significance trends were similar between the two genders, but women’s OR values for discrimination experience and direct customer confrontation were much higher than those of men.

Previous studies have examined the relationship between each category and sleep disorder; however, they have only examined factors associated with the presence or absence of general sleep disorders and insomnia. We added “sleep disorders due to one’s job,” which indicates “work-relatedness;” therefore, we analyzed work-related sleep disorder’s correlation with five variables common in previous research to better situate this study’s results within the broader research context. The present results corresponded well to the previous correlation analyses (these results are shown in the Additional file 1). Slight differences (i.e., significance at larger values of *p*) in this study’s co-analysis of additional categories may reflect sample size differences, independent variable selection, categorization differences, and differing definitions of dependent variables.

Discrimination experience significantly increased the risk of sleep disorders, including after correcting for other variables. Discrimination experience itself appears to affect general stress; gender discrimination experience leads to reduced job satisfaction, compensation satisfaction, and organizational commitment; age discrimination at work leads to disadvantages in work allocation and promotion opportunities; stress resulting from this dissatisfaction ultimately increases workers’ risk of sleep disorder [21–23].

High-risk groups in the direct customer confrontation and emotional stress categories reported sleep disorders significantly more frequently. Survey items in these categories examined if the worker frequently directly manages individuals who are not co-workers (i.e., customers, patients) and the worker’s frequency of emotional labor (which modern society is strongly focused on). Emotional labor predicts negative outcomes such as exhaustion and job dissatisfaction [24–26]. Additionally, stress level and prevalence of sleep disturbance were found to be high among female temporary workers in department stores; further, physicians and nurses (who are in close contact with patients) show high job-related stress and prevalence of sleep disturbance, corroborating this study’s results [27–29]. Requiring workers to endure negative emotions (personal implicit feelings) in order to follow company rules, and thereby distorting or exaggerating workers’ intrinsic nature, may thus lead to psychological and physical exhaustion, further provoking pathological phenomena including suicide and sleep disorder [30].

The rate of sleep disorder increased in groups with high job dissatisfaction, as found in the work dissatisfaction (detailed) and work dissatisfaction (overall) categories. This result corresponds with those of previous studies, which have additionally found that workers with low job satisfaction tended to be more stressed and more likely to experience whole-body fatigue, depression, anxiety and depression, and that low job satisfaction increases workers’ risk of insomnia [31, 32]. In contrast, the univariable analysis of organizational system dissatisfaction detected a greater OR of sleep disorder in the high-risk group; however, this difference decreased below significance after correcting for other variables. Variables that other studies defined as “organizational unfairness”(unreasonableness or unfairness of organizational policy, operational systems, or formal structure of the company) were found to be related to an increased risk of poor sleep quality with high organizational unfairness [33]. Expression of opinion difficulty was negatively correlated with workers’ risk of sleep disorder; this may reflect habitually passive workers who wish to do no more work than is required of them and are unmotivated to express their opinion, but instead prefer to passively accept information without understanding it; however, further research of this relationship is required.

Regarding high job demand, work intensity was positively correlated with workers’ risk of sleep disorder, corroborating previous results [14, 15, 34–36]. Job demand indicates work-related burden; high job demand may be a classical occupational stress factor and related to difficulty maintaining sleep and low sleep quality [37, 38].

Regarding insufficient job control, we found that difficulty controlling one’s job was negatively correlated with workers’ likelihood of reporting sleep disorder. Some previous studies have reported that workers facing greater work control difficulty reported insomnia more frequently; however, some of these studies’ hypothetical models did not achieve statistical significance [14, 39]. In contrast, some studies have found an independent effect between work control and insomnia, and have suggested that low job control affects workers’ stress more weakly and indirectly than job strain [15, 40]. This disparity may reflect differences in the measures used; however, future research should settle this dispute.

Regarding inadequate social support, workers in the high-risk group reported sleep disorder more frequently before correcting for the newly added variables; however, this difference became insignificant following correction for the newly added variables. Previous studies have also found that low social support in workplace is correlated with workers’ insomnia, citing conflict with colleagues or superiors as an important workplace stressor. In particular, individuals who work in the same workplace throughout their lifetime were found to prioritize interpersonal working relationships [14, 15].

Regarding job insecurity, the high-risk group reported sleep disorder more frequently, corroborating previous study results [14, 41]. Job insecurity indicates one’s stability in one’s position at work, capturing employment opportunities as well as instability. In South Korea, for example, most employees indicated exposure to serious threats to their psychological health from external factors such as downsizing and employment instability due to the rapidly changing socioeconomic situation since the financial crisis of 1997 [17].

Workers reporting a lack of reward were more likely to report sleep disorder. Inadequate compensation is a concept taken from the Effort-Reward-Imbalance (ERI) model: high effort and low compensation leads to high stress. In the present context, high ERI increased workers’ insomnia, corroborating previous studies [14, 15, 36].

Workers who reported working over 45 h per week and shift workers were significantly more likely to report sleep disorder. Numerous studies have also found this association, and have suggested desynchronization of circadian rhythms due to irregular sleep-wake schedule as the main cause of such work-induced sleep disorders [4, 32, 42, 43]. Other studies examining working hours per week have suggested a plausible causal pathway between overtime working hours and sleep disorder, mediated by poor recovery after work due to inadequate free time, and have therefore proposed the necessity of sufficient time for recovery from fatigue [34, 38, 44].

Earlier research has not fully established a cause-effect mechanism between job-related stress and sleep disorder; however, some studies have reported these variables to be mediated by increased cortisol levels [36, 45, 46]. Increased cortisol secretion and hyperactivity of the sympathetic nervous system following activation of the hypothalamic-pituitary-adrenal axis is a relatively direct biological response to stress and may elicit various physiological responses [47, 48]. More indirectly, behaviors detrimental to health (i.e., smoking, consuming alcohol) may manifest in response to stress [23]. Stressful life events may powerfully affect the development of insomnia; similarly, minor stressors (e.g. Job strain) in daily life are also correlated with sleep disorder [49]. Further, psychosocial factors are more strongly correlated with consistent insomnia than physical health problems [50]. Research thus supports the correlation between stress and sleep disorder. Future research should promote acceptance of job-related stress as importantly affecting sleep disorder, illuminate the disorder’s causal mechanism more clearly, and intervene to prevent the disorder’s development.

This study possesses the following limitations. First, measurement involved the individuals taking the survey subjectively diagnosing themselves with sleep disorder. The KWCS was not specifically designed to measure stress and sleep disorder; responders therefore subjectively judged if they had sleep disorder. This does not meet the diagnostic criteria of sleep disorder set out by the World Health Organization (WHO), the International Classification of Diseases (ICD-10-CM), or the Diagnostic and Statistical Manual of Mental Disorders (DSM-5) by American Psychiatric Association (APA); it also diverges from the International Classification of Sleep Disorders (ICSD-2, 2005) of the American Academy of Sleep Medicine (AASM). The validity of sleep disorder diagnosis in this study may therefore have been limited; the survey also did not specifically examine sleep quality and quantity. Second, this study used a cross-sectional research design; this prevents valid inferences regarding variables’ chronology and causal relationships. Third, we did not use a standardized tool to measure job-related stress factors. Jang et al. developed the Occupational Stress Scale for Korean Employees; however, this study combined this measure’s questions with others taken from the KWCS. Additionally, the author altered some survey items following comparison of the two surveys; the measure of job stress was therefore non-standardized. Nonetheless, reliability analysis of the survey yielded Cronbach’s alpha values exceeding 0.6 in all categories, exceeding adequacy cut-offs and indicating acceptable response consistency. Fourth, the participants were current workers; workers who had retired due to accident, disease or health deterioration were not included in the analysis. The results may therefore reflect a “healthy worker” effect, leading to a general underestimation of sleep disorders’ prevalence. In contrast, the results may also reflect recall bias: individuals personally experiencing sleep disorders may have overestimated stress level as the measure was self-administered.

Despite these limitations, the results of this study remain significant. This study identified and analyzed significant stress factors not analyzed in previous research, and found them correlated with job-related stress and sleep disorder; this deepens the understanding of the existing factors, and extends the range of known factors affecting job-related stress. In particular, the present results indicate that experience of discrimination, direct customer confrontation, and emotional labor significantly elevates workers’ sleep disorder risks; therefore, these factors merit additional attention. In addition, regarding discrimination experience, the OR of sleep disorder was approximately 1.7 times higher for women than for men; hence, some gender differences in exposure and risk of stress exist. Women are more vulnerable to stress from discrimination and disease. Questions regarding discrimination should be subdivided into discrimination by gender, age, education level, region, and employment type. Moreover, items pertaining to dealing with emotional labor should be added to the existing stress measurement tools. Further, previous studies have examined only insomnia or sleep disorder; in contrast, this study examined whether workers believed their symptoms to be work-related. This more specifically captures the correlation between workers’ evaluations of sleep disorder and job-related stress. Doctors should consider that workers’ sleep disorders may be associated with the stress they experience in their workplace, rather than with a personal problem. An objective diagnosis and treatment system should therefore be established to more accurately explore the role of work-related psychosocial factors. Finally, this study used data from a large-scale survey examining workers from all over Korea; this raises the likelihood that the present results are representative.

Sleep disorders including insomnia may accompany various psychiatric disorders, or occur as initial symptoms of a disorder or constitute part of a latent period of further disorder. Further, sleep disorder is closely correlated with various physiological diseases (e.g., cardiovascular diseases, chronic pain, gastrointestinal diseases) [51]. The present results confirm that job-related stress significantly predicts sleep disorder; interventions should therefore reduce workplace stress by controlling workers’ labor or relocating stressed workers. Further, social efforts should aim to create workplace cultures that allocate recreational or break time in order to alleviate accumulated worker stress.

## Conclusions

In conclusion, this study found a strong correlation between sleep disorder and relational and organizational job stress factors. Sleep disorder may lead to large decreases in workers’ quality of life and work efficiency. Awareness and interventions are therefore required to reduce workplace stress; further research of this topic is also required.

## Additional file

Additional file 1: Table S4. Odds ratio of selected variables and Sleep disorder. (XLSX 14 kb)
